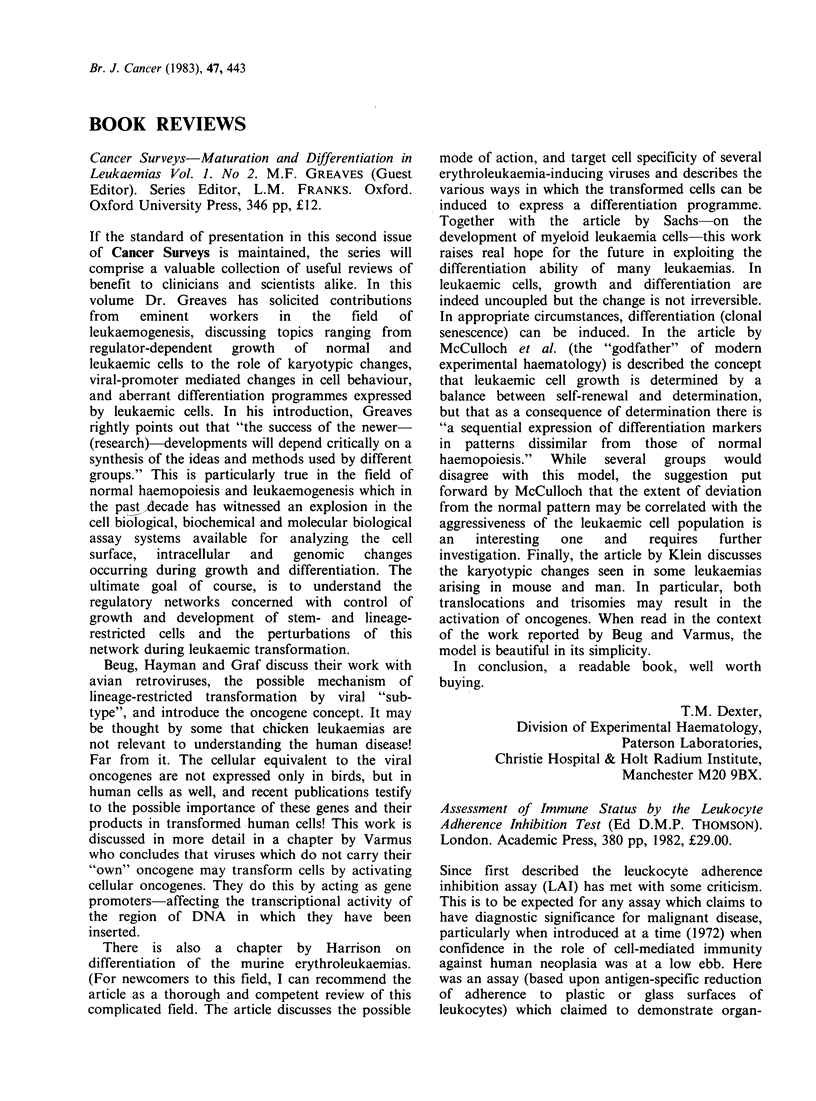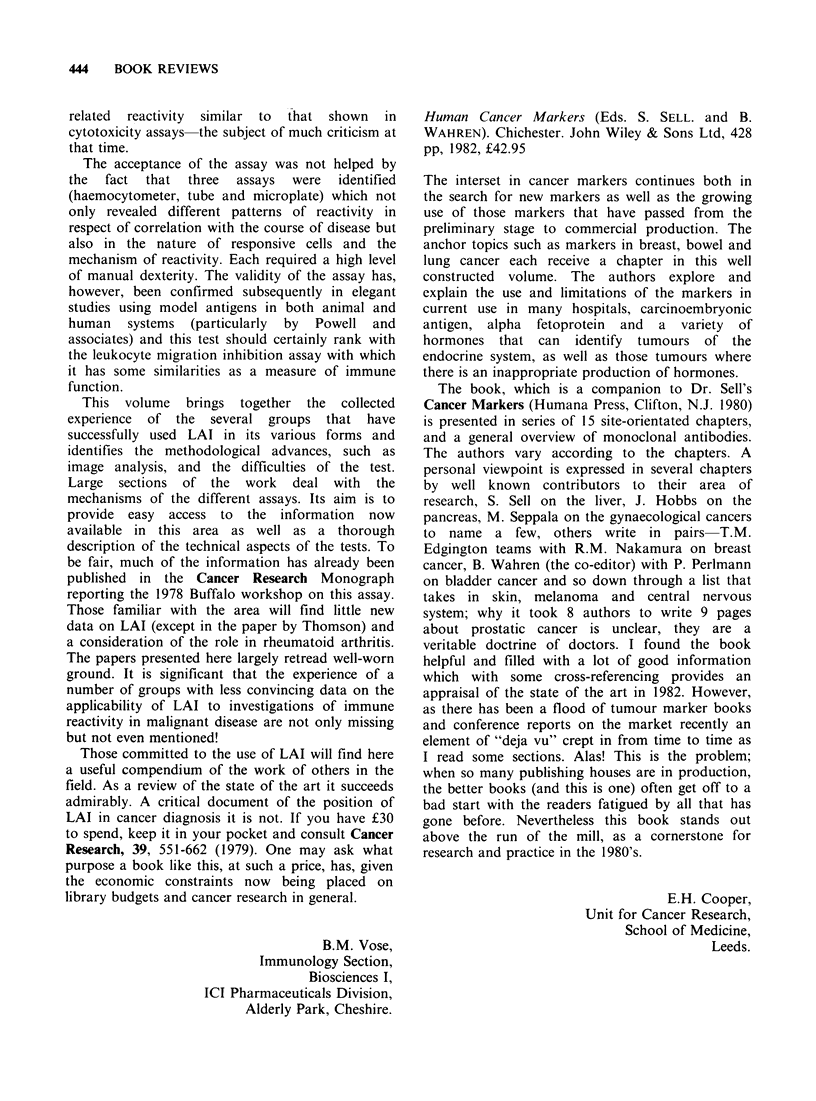# Assessment of Immune Status by the Leukocyte Adherence Inhibition Test

**Published:** 1983-03

**Authors:** B.M. Vose


*Assessment of Immune Status by the Leukocyte Adherence Inhibition Test* (Ed D.M.P. THOMSON). London. Academic Press, 380 pp, 1982, £29.00.

Since first described the leuckocyte adherence inhibition assay (LAI) has met with some criticism. This is to be expected for any assay which claims to have diagnostic significance for malignant disease, particularly when introduced at a time (1972) when confidence in the role of cell-mediated immunity against human neoplasia was at a low ebb. Here was an assay (based upon antigen-specific reduction of adherence to plastic or glass surfaces of leukocytes) which claimed to demonstrate organrelated reactivity similar to that shown in cytotoxicity assays—the subject of much criticism at that time.

The acceptance of the assay was not helped by the fact that three assays were identified (haemocytometer, tube and microplate) which not only revealed different patterns of reactivity in respect of correlation with the course of disease but also in the nature of responsive cells and the mechanism of reactivity. Each required a high level of manual dexterity. The validity of the assay has, however, been confirmed subsequently in elegant studies using model antigens in both animal and human systems (particularly by Powell and associates) and this test should certainly rank with the leukocyte migration inhibition assay with which it has some similarities as a measure of immune function.

This volume brings together the collected experience of the several groups that have successfully used LAI in its various forms and identifies the methodological advances, such as image analysis, and the difficulties of the test. Large sections of the work deal with the mechanisms of the different assays. Its aim is to provide easy access to the information now available in this area as well as a thorough description of the technical aspects of the tests. To be fair, much of the information has already been published in the **Cancer Research** Monograph reporting the 1978 Buffalo workshop on this assay. Those familiar with the area will find little new data on LAI (except in the paper by Thomson) and a consideration of the role in rheumatoid arthritis. The papers presented here largely retread well-worn ground. It is significant that the experience of a number of groups with less convincing data on the applicability of LAI to investigations of immune reactivity in malignant disease are not only missing but not even mentioned!

Those committed to the use of LAI will find here a useful compendium of the work of others in the field. As a review of the state of the art it succeeds admirably. A critical document of the position of LAI in cancer diagnosis it is not. If you have £30 to spend, keep it in your pocket and consult **Cancer Research, 39**, 551-662 (1979). One may ask what purpose a book like this, at such a price, has, given the economic constraints now being placed on library budgets and cancer research in general.

B.M. Vose,
Immunology Section,
Biosciences I,
ICI Pharmaceuticals Division,
Alderly Park, Cheshire.